# The Dental Cosmos*This was written for the September number, but was overlooked.

**Published:** 1862-10

**Authors:** 


					﻿THE DENTAL COSMOS.*
This journal began its fourth volume with the August number,
without change of character, publisher, or editors. The publish-
er’s announcement—now that he is not a member of a firm, and
can, therefore, say just what he pleases—is as modest as himself.
One of the editors gives us a “ retrospect ” of his three years’ la-
bor, which is quite readable, although lengthy, and would be
highly satisfactory, were it not that the reader is constantly im-
pressed with the idea that the writer is in misery. Has anything
hurt his feelings ? It is to be hoped not, for a man’s feelings are
the tenderest part of him. These who “ give their thoughts to
the public ” should take plenty of fresh air, eat healthy food, and
sleep regularly and enough; for when a man’s brain is wearied,
his nerves unstrung, and especially when an undigested fragment
of a sour apple is trying to force its way through the pylorus, he
imagines every man hostile who looks at him, though his foes are
as unreal as the “snakes” in the drunkard’s boots. (Having
retired from general practice, nothing is charged for this prescrip-
tion, and it is intended “ for the good of all concerned.”) The
Cosmos is ever a welcome visitor ; and we hope our readers will
conclude the Register looks lonely by itself, and take the Cosmos
to afford it company. A live dentist needs at least two living
journals.	W.
*This was written for the September number, but was overlooked.
				

